# Association between Sleep-Disordered Breathing during Pregnancy and Maternal and Fetal Outcomes: An Updated Systematic Review and Meta-Analysis

**DOI:** 10.3389/fneur.2018.00091

**Published:** 2018-05-28

**Authors:** Liwen Li, Kena Zhao, Jin Hua, Shenghui Li

**Affiliations:** ^1^MOE – Shanghai Key Laboratory of Children’s Environmental Health, Shanghai Jiao Tong University School of Medicine, Xinhua Hospital, Shanghai, China; ^2^School of Public Health, Shanghai Jiao Tong University School of Medicine, Shanghai, China; ^3^Shanghai First Maternity and Infant Hospital Corporation, Tongji University, Shanghai, China

**Keywords:** sleep-disordered breathing, maternal and child health, meta-analysis, pregnancy complication, gestational diabetes mellitus

## Abstract

**Background:**

Due to the high prevalence in pregnant women and potential association with pregnancy complications or perinatal outcomes, sleep-disordered breathing (SDB) has become an increasing concern.

**Methods:**

Pubmed and Embase were retrieved from inception until 2017 to conduct a meta-analysis to explore the association of SDB and several outcomes during gestation. A stratified analysis differentiated by the type of SDB [snoring alone/obstructive sleep apnea (OSA)] was also performed. Pooled odds ratios were produced for binary outcomes. Weighted mean differences were also produced for continuous outcomes. Sensitivity analysis was performed to identify the impact of individual studies on summary results and estimation of publication bias was performed by funnel plot.

**Results:**

35 studies with a total of 56,751,837 subjects were included. SDB during pregnancy was associated with a significantly increased risk of gestational diabetes mellitus (GDM), pregnancy-induced hypertension (PIH), and preeclampsia (PEC), but not significantly associated with fetal maternal outcomes, namely APGAR score and birth weight. Moreover, OSA was linked with an increasing risk of GDM, PIH, PEC and preterm birth while snoring appeared to increase the risk of GDM, PIH, and PEC.

**Conclusion:**

The finding provided potential evidence for association between SDB and adverse perinatal outcomes. SDB increased the risk of some pregnancy complications while its influence to fetal outcomes was not clear.

## Introduction

Sleep-disordered breathing (SDB) or sleep-related breathing disorders are characterized by abnormalities of respiration during sleep. SDB can be further grouped into obstructive sleep apnea (OSA), central sleep apnea disorders, sleep-related hypoventilation disorders, and sleep-related hypoxemia disorder (1). A published study indicated that SDB was highly prevalent in pregnant women for substantial physiological and hormonal changes during the gravid stage (2). Meanwhile, it has been confirmed that pregnant women are more vulnerable to OSA than non-pregnant women (3). A study conducted on 500 females suggested that the prevalence of snoring increased from 7.9 to 21.2% through the first to the third trimester of pregnancy (4).

The considerable prevalence of SDB in pregnant women has drawn increasing attention. As suggested by recent studies, SDB has potentially increased the risk of advanced pregnancy complications such as gestational diabetes mellitus (GDM), pregnancy-induced hypertension (PIH), and preeclampsia (PEC) (5–7). These pregnancy complications can be strongly related to several adverse maternal outcomes. For instance, GDM has been recognized as a risk factor for neonatal hypoglycemia, premature delivery and fetal macrosomia (8–10). Besides, SDB may be associated with preterm birth (PTB) and low birth weight (BW). It was suggested by several studies that both snoring and OSA were linked with an increased risk of PTB, a major cause of infant mortality (6, 11–13). Low BW impacted neonatal mortality in a similar way (14). Also, it was indicated that gestational SDB was likely to affect the neurobehavioral development in infants (15).

In recent years, researchers attempted to clarify the association between SDB and maternal–fetal outcomes. However, the corresponding results were not conclusive and some contradictions have been found in the current literature. For instance, a recent meta-analysis indicated a significant impact of SDB on GDM, PIH, and BW (16) while another study denied such an impact (17). Besides that, SDB was reported to induce the elevation of circulating inflammatory markers and this trend can be used to predict PTB (18), but this conclusion was challenged by a study which revealed no significant association between SDB and PTB (17). Apart from these controversies, the power of some studies was limited by other factors such as the small sample size (19). For this sake, we designed and conducted this meta-analysis to examine whether SDB was associated with adverse pregnancy complications and maternal–fetal outcomes.

## Materials and Methods

### Literature Search Strategy

We commenced our study by systematically searching medical databases including PubMed and Embase for relevant literatures from inception until 2017.

A comprehensive search strategy was applied with the following terms as keywords: “sleep apnea syndromes” or “snoring” or “obstructive sleep apnea” and “pregnancy” or “pregnant women” or “infant” and “APGAR score” or “gestational diabetes.” Bibliographies of retrieved articles were also reviewed and manually searched to avoid any potential omissions.

### Inclusion Criteria

The retrieved literatures to be included in our study must meet all the following criteria: (1) observational studies with pregnant women as subjects; (2) observational (prospective or cross-sectional) studies without any intervention; (3) SDB was diagnosed as either snoring or OSA using overnight sleep monitoring (20), Berlin Questionnaire (21), or diagnostic criteria illustrated in International Classification of Sleep Disorders Third Edition (ICSD-3); (4) at least one of the pregnancy complications including GDM, PIH, PEC, or fetal outcomes including BW, APGAR (5 min), and PTB was assessed in the study; and (5) Sufficient data could be derived for evidence synthesis and statistical analysis. As for the studies from the same researchers, the larger samples one will be included. The precision is driven primarily by the sample size, with larger studies yielding more precise estimates of the effect size.

According to ICSD-3, diagnosis of OSA is confirmed by obstructive events (apneas, hypopnea, and respiratory events related to arousals) on polysomnography of ≥15 events/h or ≥5/h in a patient who reports symptoms including unintentional sleep episodes during wakefulness, daytime sleepiness, unrefreshing sleep, fatigue, insomnia, waking up breath holding, gasping, or choking; or the bed partner describing loud snoring, breathing interruptions, or both during patient’s sleep. Snoring is one symptom of breathing-related sleep disorder which leads to excessive sleepiness or insomnia due to a sleep-related breathing condition (e.g., obstructive or central sleep apnea syndrome) (22).

### Data Extraction

Data were extracted from the included studies as follows: name of author, publication year, country of origin, study type, sample size, age, and body mass index (BMI) of study subjects as well as the clinical outcomes including GDM, PIH, PEC, APGAR, BW, and PTB as mentioned above. Two researcher extracted data from original studies independently and any discrepancy between two reviewers would be resolved by a third reviewer.

### Statistical Analysis

Data were synthesized to compare the clinical outcomes of pregnant women with and without SDB. For binary outcomes, namely GDM, PIH, PEC, and PTB, odds ratios (ORs) and 95% confidence intervals (CIs) were used to evaluate the effect size. The weighted mean differences (WMDs) and their 95% CIs were applied for continuous outcomes BW and APGAR.

Adjusted odds ratio (aOR) is OR extracted from included study. Raw odds ratio (rOR) is OR calculated when only raw data was accessible in previous studies.

All analyses were conducted by R version 3.3.3. Investigation of heterogeneity among eligible studies was carried out according to Cochran’s *Q*-statistic and *I*^2^ test. Significant heterogeneity was present when *P*-value of Cochran’s *Q*-test was less than 0.05 or *I*^2^ proved to be larger than 50%. Under such a circumstance, a random-effects model (*DerSimonian-Laird method*) was applied to replace the fixed-effects model (*Mantel-Haenszel method*) to improve the accuracy of research. Additionally, a sensitivity analysis was performed by removing each study to evaluate how individual studies impacted on the summary statistic produced by our meta-analysis. The reappraised results after exclusion of each study were compared with original results to estimate the reliability of our analysis. Finally, publication bias was assessed by funnel plot.

## Results

### Characteristics of Studies

**Figure 1:**
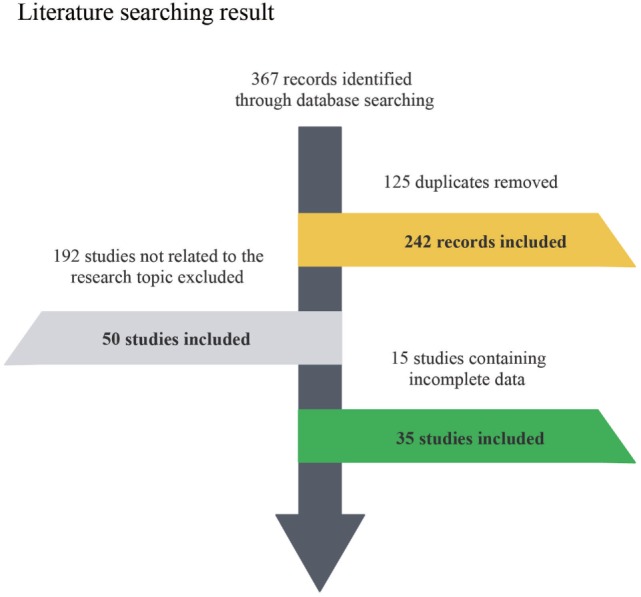
Flow diagram of included and excluded studies.

The flow chart indicates the entire process of literature search, identification and screening (Figure 1). The search range was from inception to June 2017. A total of 367 records were identified initially through database searching from PubMed and Embase and 125 duplicates were later removed. Since 192 of the remaining 242 studies were not related to the research topic, 50 studies were retained in the second step. Another 15 studies without sufficient data or full-text content were removed afterward. The remaining 35 studies with a total of 56,751,837 subjects were finally identified as eligible (4–7, 11, 12, 19, 23–50), covering a period from 1996 to 2017. For all included studies, baseline characteristics and target outcomes of the enrolled studies were summarized in Table 1.

**Table 1:** Characteristics of Studies Included in meta-analysis.

Author, year, country	Study type	Patient type	Number		Age (years)		BMI (kg/m^2^)		Outcomes
		
			+	−	+	−	+	−	
Loube, 1996, USA	Prospective	Snoring	49	301	27.0 (9.0)	25.0 (6.0)	29.8 (4.0)	28.0 (4.0)	④
Franklin, 2000, Sweden	Cross-sectional	Snoring	113	389	29.8 (5.4)	28.4 (4.7)	–	–	②③
Koken, 2007, Turkey	Prospective	Snoring	40	43	30.8 (5.0)	25.6 (3.9)	30.5 (6.4)	25.5 (5.1)	④⑤
Perez-Chada, 2007, Argentina	Cross-sectional	Snoring	156	291	28.9 (6.9)	27.5 (7.9)	27.2 (4.3)	25.9 (3.0)	②④
Sahin, 2008, Turkey	Prospective	OSA	4	31	34.8 (3.3)	30.7 (5.1)	37.5 (8.4)	30.6 (6.7)	④
Ursavas, 2008, Turkey	Prospective	Snoring	55	414	26.7 (5.1)	25.3 (4.8)	23.6 (4.1)	22.8 (3.8)	②③
Bourjeily, 2010, USA	Cross-sectional	Occasional snoring	133	480	29.6 (5.9)	28.2 (6.0)	32.1 (6.3)	32.1 (6.3)	①②
		Frequent snoring	331		30.3 (6.0)		32.1 (6.3)		①
Louis, 2010, USA	Prospective	Snoring	57	114	30.0 (6.0)	23.0 (5.0)	46.0 (13.0)	22.0 (2.2)	①②③④⑥
Qiu, 2010, USA	Prospective	Snoring	89	1,169	33.3 (4.4)	33.3 (4.4)	23.5 (4.5)	23.5 (4.5)	①
Ayrim, 2011, Turkey	–	Snoring	41	158	27.4 (6.7)	27.4 (6.7)–	28.2	26.7	⑤⑥
Micheli, 2011, Greece	Prospective	Occasional snoring	151	892	–	–	25.1 (4.8)	23.9 (4.8)	②
		Severe snoring	48		–		26.8 (6.1)		④
Higgins, 2011, USA	Prospective	OSA	1,343	2,731	33.0 (1.8)	32.0 (1.5)	32.0 (1.5)	27.0 (1.0)	③④
Olivarez, 2011, USA	Cross-sectional	OSA	56	164	29.5	27.9	33.4	29.5	①②③
Tauman, 2011, Israel	Preliminary	Snoring	48	74	30.7 (4.5)	30.3 (4.6)	22.9 (3.6)	21.5 (3.6)	⑤⑥
Bourjeily, 2012, USA	–	OSA	38	722	29.1 (6.1)	29.1 (6.1)	26.0 (6.2)	26.0 (6.2)	②
Chen, 2012, Taiwan	Cross-sectional	OSA	791	3,955	–		–		①③⑥
Facco, 2012, USA	Cross-sectional	Mild SDB	34	83	31.8 (6.1)	31.0 (5.3)	39.6 (12.1)	30.5 (10.1)	①⑥
		Considerable SDB	26		32.5 (4.9)		44.2 (11.0)		⑥
Louis, 2012, USA	Prospective	OSA	26	135	30.0 (6.4)	27.3 (5.9)	48.3 (11.8)	39.1 (6.2)	①②③④⑥
Tauman, 2012, Israel	–	Chronic snoring	20	168	32.6 (4.0)	31.4 (4.6)	23.1 (3.9)	21.9 (3.4)	④⑤
		New-onset snoring	58		30.4 (4.9)		22.9 (3.5)		③
O’Brien, 2012, USA	Prospective	Snoring	584	1,128	30.3 (5.9)	29.4 (5.8)	29.3 (8.6)	25.0 (6.1)	②③④
Owusu, 2013, USA	Cross-sectional	Snoring	53	167	29.1 (5.5)	28.8 (5.8)			③④
Fung, 2013, Australia	Prospective	OSA	14	27	36.0 (4.4)	33.4 (4.8)	35.1 (5.4)	31.0 (8.9)	①②③④
Ko, 2013, Korea	Prospective	OSA	89	187	32.7	32.8	27.2	25.8	②③
O’Brien, 2013, USA	Prospective	Snoring	586	1,087	29.8 (5.8)	29.8 (5.8)	26.5 (7.2)	26.5 (7.2)	④⑤
Antony, 2014, USA	Prospective	OSA	456	1,053	29	28.7	31.9	28.8	①②③
Facco, 2014, USA	Prospective	Mild SDB	40	127	33.0 (5.9)	33.0 (5.9)	32.8 (8.7)	32.8 (8.7)	①③⑥
		Considerable SDB	20		33.0 (5.9)		32.8 (8.7)		①③⑥
	Prospective	Mild SDB	45	67	–	–	–	–	①③⑥
		Considerable SDB	16		–		–		①③⑥
Louis, 2014, USA	Cross-sectional	OSA	16,735	55,765,230	–	–	–	–	①②③⑥
Sarberg, 2014, Sweden	Prospective	Gestational snoring	45	268	29.4 (4.08)	30.2 (4.33)	24.4 (3.3)	23.6 (3.8)	②
		Habitual snoring	27		29.6 (5.7)		26.4 (5.0)		②
Sharma, 2015, India	Prospective	Snoring^a^	53	156	–	–	–	–	①②
Bassan, 2016, Israel	Prospective	Snoring	11	33	32.3 (2.8)	32.5 (4.7)	30.5 (6.7)	23.0 (2.7)	①④⑤
Ge, 2016, China	Prospective	Chronic snoring	361	2,568	26.5 (3.5)	36.0 (3.5)	–	–	①②③⑥
		Habitual snoring	150		27.2 (4.6)		–	–	①③⑥
Spence, 2017, USA	Retrospective	OSA	266	304,735	26.72 (5.22)	30.68 (6.38)	–	–	①②③⑥
Facco, 2017, USA	Prospective	SDB	114	3,017	27.1 (5.5)	29.2 (5.8)	–	–	①②③
Bin, 2016, Australia	Popular-base	Sleep apnea	519	635,708	–	–	–	–	①②⑥
Tauman, 2015, Israel	–	SDB	18	56	33.1 (3.7)	32.7 (4.6)	25.3 (4.3)	21.9 (2.5)	⑤

*“+” represents the study objectives with certain symptom, while “−” represents those without that symptom type*.

*Clinical outcomes ① GDM: gestational diabetes mellitus ② PIH: pregnancy-induced hypertension ③ PEC: preeclampsia ④ BW: birth weight (g) ⑤ APGAR (5min): APGAR score ⑥ PTB: premature birth*.

*^a^Snoring includes snoring once, twice, and three times or more, respectively*.

*OSA, obstructive sleep apnea; SDB, sleep-disordered breathing*.

### Gestational Diabetes Mellitus

**Figure 2:**
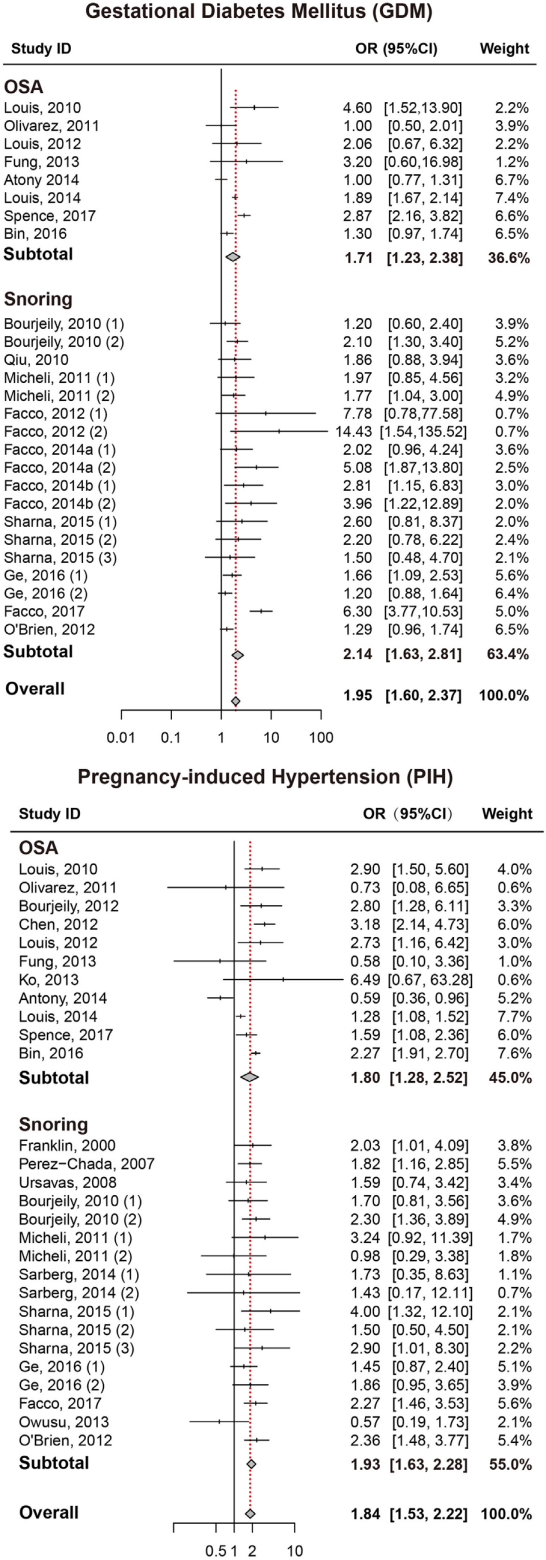
Forest plots of the association between GDM/PIH and SDB (snoring and OSA). Results are expressed as OR and 95% CI. GDM, gestational diabetes mellitus; PIH, pregnancy-induced hypertension; SDB, sleep-disordered breathing; OSA, obstructive sleep apnea; OR, odds ratio; CI, confidence interval. The numbers after authors were used to distinguish different groups within one trial.

As presented in Figure 2, pregnant women with SDB were associated with a significant increased risk of GDM compared to those without SDB (OR = 1.95, 95% CI = 1.60–2.37). We also performed a stratified analysis based on the type of SDB (snoring or OSA; Table 2). Women with snoring were associated with an increased risk of GDM (OR = 2.14, 95% CI = 1.63–2.81), and such an association was also significant between OSA and GDM (OR = 1.71, 95% CI = 1.23–2.38).

**Table 2:** Overall results of pregnancy complication and fetal outcomes.

Outcomes	Snoring			OSA			SDB		
	*I*^2^	*P*-value	OR (95% CI)	*I*^2^	*P*-value	OR (95% CI)	*I*^2^	*P*-value	OR (95% CI)
Gestational diabetes mellitus	65%	<0.01	2.14 (1.63, 2.81)	82%	<0.01	1.71 (1.23, 2.38)	72%	<0.01	1.95 (1.60, 2.37)
Pregnancy-induced hypertension	0%	0.72	1.80 (1.28, 2.52)	83%	<0.01	1.93 (1.63, 2.28)	62%	<0.01	1.84 (1.53, 2.22)
Preeclampsia	58%	<0.01	1.87 (1.27, 2.75)	78%	<0.01	2.63 (1.87, 3.70)	71%	<0.01	2.19 (1.70, 2.82)
Birth weight	33%	0.22	26.54 (−52.53, 105.60)	93%	<0.01	−47.46 (−242.09, 147.16)	91%	<0.01	16.75 (−53.53, 87.03)
APGAR score	0%	0.75	−0.02 (−0.06, 0.02)	Not applicable		−1.00 (−2.37, 0.37)	0	0.75	−0.02 (−0.06, 0.02)
Preterm birth	22%	0.22	1.22 (0.87, 1.70)	90%	<0.01	1.75 (1.21, 2.55)	71%	<0.01	1.47 (1.14, 191)

*Results are expressed as OR with 95% CI, birth weight, and APGAR are expressed with WMD with 95% CI*.

### Pregnancy-Induced Hypertension

Pregnant women with SDB appeared to take significantly higher risk of PIH compared to those without SDB (OR = 1.84, 95% CI = 1.53–2.22; Figure 2; Table 2). Stratified analysis indicated that both snoring and OSA were significantly associated with an increased risk of PIH (OR = 1.93, 95% CI = 1.63–2.28; OR = 1.80, 95% CI = 1.28–2.52, respectively).

### Preeclampsia

**Figure 3:**
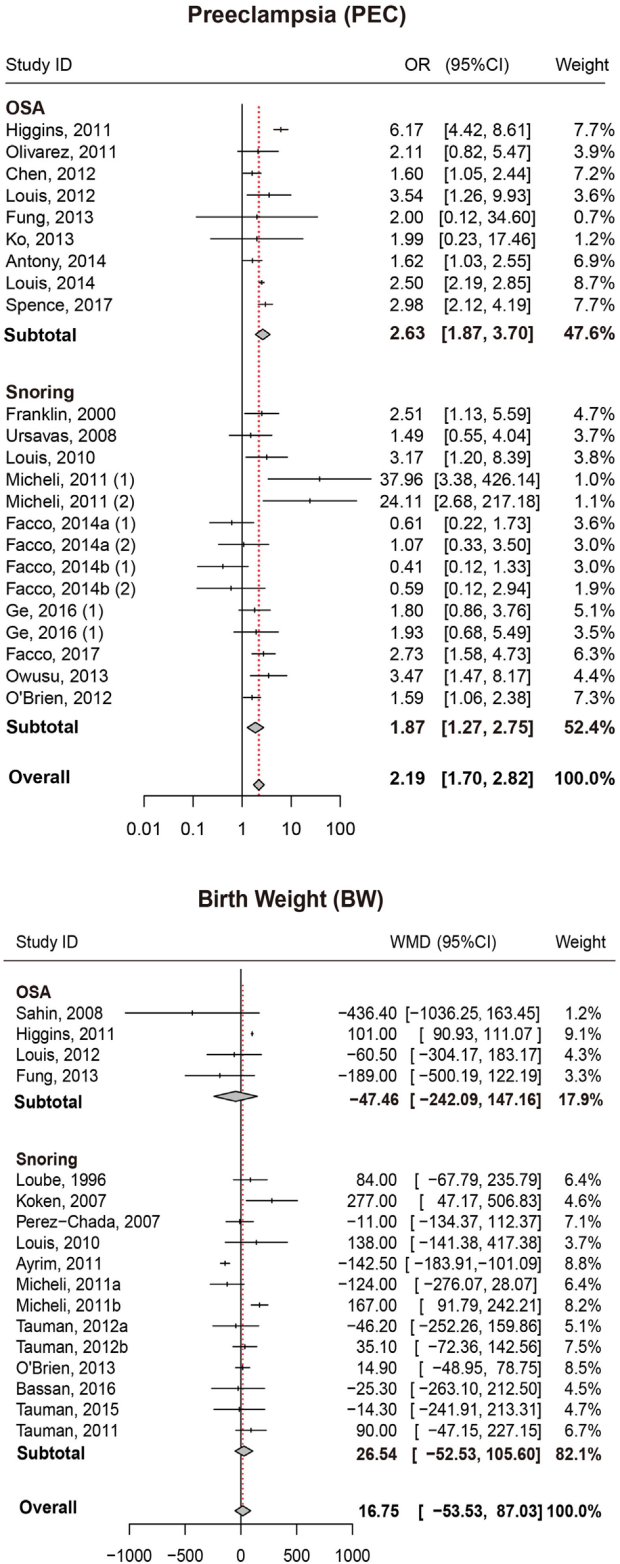
Forest plot of the association between PEC/BW and SDB (snoring and OSA). Results are expressed as OR or WMD with 95% CI. PEC, preeclampsia; BW, birth weight; SDB, sleep-disordered breathing; OSA, obstructive sleep apnea; OR, odds ratio; WMD, weighted mean difference; CI, confidence interval. The numbers after authors were used to distinguish different groups within one trial.

We also attempted to discover whether SDB was associated with the risk of PEC and whether this association differed significantly between different types of SDB (snoring or OSA). Overall, there was significant association between SDB and PEC (OR = 2.19, 95% CI = 1.70–2.82). Moreover, stratified analysis revealed that pregnant women with OSA and snoring all appeared to have an increased risk of PEC (OR = 2.63, 95% CI = 1.87–3.70; OR = 1.87, 95% CI = 1.27–2.75, respectively; Figure 3; Table 2).

### Birth Weight

No significant difference in BW between pregnant women with SDB and those without SDB was observed in our study. Results of stratified analysis by the type of SDB were consistent with the overall analysis (Figure 3; Table 2).

### APGAR (5 min)

**Figure 4:**
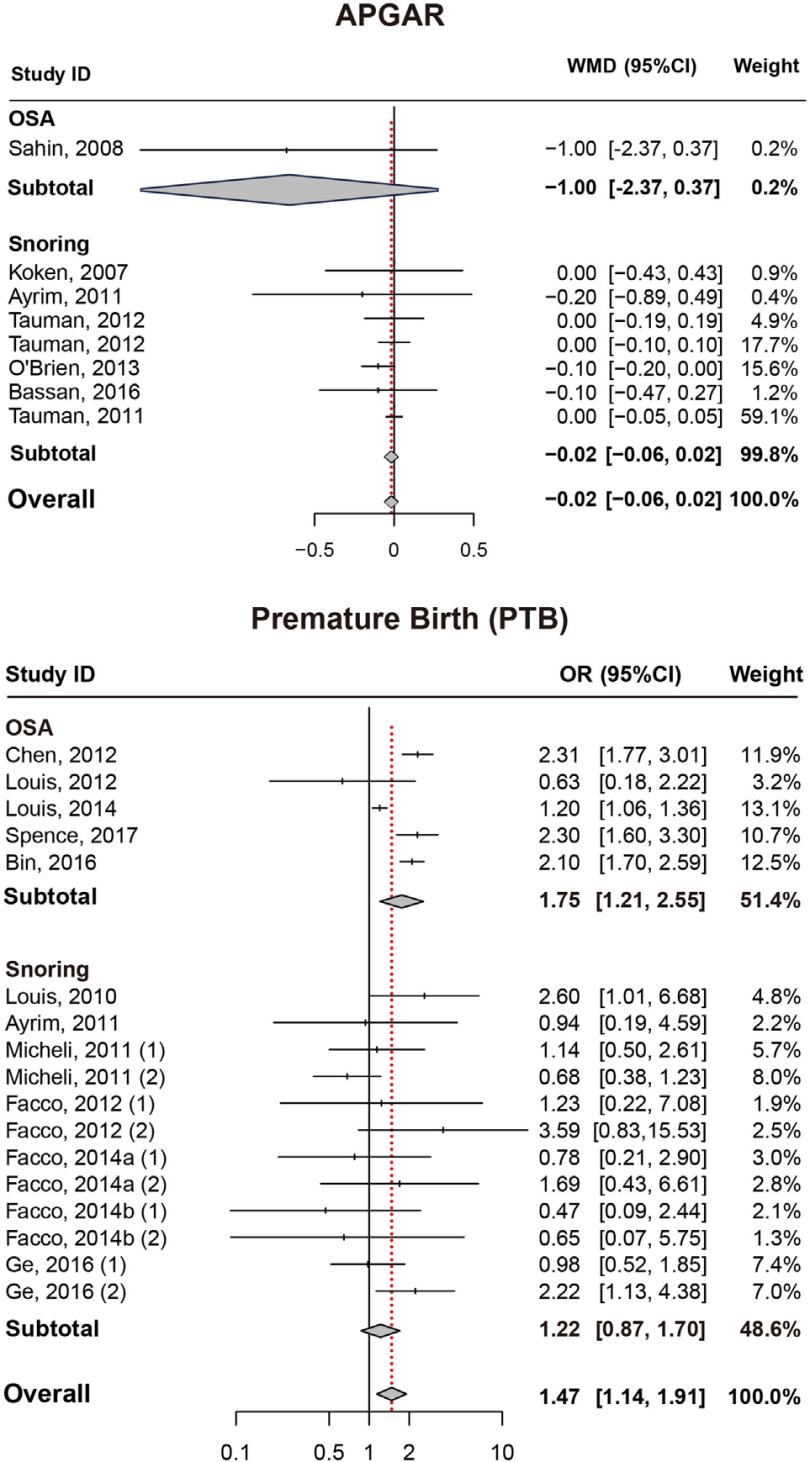
Forest plot of the association between APGAR/PTB and SDB (snoring and OSA). Results are expressed as OR or WMD with 95% CI. APGAR, APGAR score; PTB, preterm birth; SDB, sleep-disordered breathing; OSA, obstructive sleep apnea; OR, odds ratio; WMD, weighted mean difference; CI, confidence interval. The numbers after authors were used to distinguish different groups within one trial.

Results also suggested no significant difference in APGAR (5 min) score between pregnant women with SDB or not (Figure 4; Table 2).

### Preterm Birth

As presented in (Figure 4; Table 2), significant association between SDB and PTB was existed (OR = 1.47, 95% CI = 1.14–1.91). Besides, pregnant women with OSA took high risk of PTB (OR = 1.75, 95% CI = 1.21–2.55) while no significance was identified with snoring in pregnant women (OR = 1.22, 95% CI = 0.87–1.70).

### Sensitivity Analysis and Publication Bias

Sensitivity analysis was carried out to confirm whether the above results were significantly influenced by a set of studies. As displayed in Figures S1 and S2 in Supplementary Material, estimates with respect to all outcomes did not differ substantially. We also conducted statistical tests for assessing the risk of publication bias and small-study effects. The funnel plots were displayed in Figures S3–S5 in Supplementary Material. Since the included studies roughly appeared a symmetry distribution and no abnormal point was shown in the plots, we concluded that there was no significant publication bias with respect to each outcome.

## Discussion

Pregnancy is a unique physiological stage in which women experience remarkable changes in hormone secretion, respiration physiology, cardiovascular function and sleep. Several studies have suggested that the incidence of SDB increased with the progression of pregnancy. Thus, we speculated that the above changes were likely to have some impact on the pathology of SDB. For instance, a high estradiol levels during pregnant may cause a narrowing of the upper airways and this could lead to snoring. It was also reported in a previous survey that weight gain might be another conceivable mechanism to explain why women with SDB during pregnancy experienced significant increase (24). As suggested by the famous Developmental Origins of Health and Disease, adverse maternal events during the early course of pregnancy may produce permanent and structural changes in infants through the process of Early Life Programming and Imprinting (51). As a result, identifying key adverse maternal events plays a critical role in maternal and infant care. In our study, the exploration of the association between SDB during pregnancy and perinatal outcomes is essential.

The present study conducted a meta-analysis to detect the association between SDB during pregnancy and perinatal outcomes. Unlike previous studies, both pregnancy complications and fetal–maternal outcomes were investigated in our study: GDM, PIH, PEC, BW, APGAR score, and PTB (4, 31). We discovered that gestational SDB appeared to be associated with some pregnancy complications, specifically with an increased risk of GDM, PIH, PEC, and PTB. As suggested by our study, there was no association between gestational SDB and fetal outcomes (APGAR scores, BW). A stratified analysis by the type of SDB (snoring/OSA) further suggested that pregnant women with snoring alone were associated with an increased risk of GDM, PIH, and PEC, the similar association existed in OSA. Our meta-analysis updated the included studies and supported the conclusions from Ding 2014 (16) article. Besides, our meta-analysis made a distinction between snoring and OSA while Ding 2014 article did not. In addition, we also updated data when it comes to comparison with prior studies. However, our study did not show significant association between snoring and the risk of PTB, but we confirmed that pregnant women with OSA exhibited a higher risk of PTB.

The majority of current studies investigated the epidemiological features of SDB and their potential association with maternal health. However, studies rarely explained the corresponding mechanism underpinned the potential association. For example, a large scale studies conducted in the USA revealed that gestational OSA accompanied with obesity may increase its association with GDM, PIH, PEC, and PTB (38), but in this typical cross-sectional study the possible causal relationship between OSA and pregnancy complications was not particularly explained. To our knowledge, SDB can induce intermittent hypoxia and sleep fragmentation which may cause sympathetic activation, oxidative stress and inflammation (52). Sympathetic activation may further trigger blood pressure changes, alter glucose homeostasis and induce insulin resistance, eventually causing hypertension and diabetes (53). Similarly, both oxidative stress and inflammation were also thought to be responsible for insulin resistance and gestational diabetes (54). Furthermore, SDB during pregnancy experiences significant increase in the chest load when they begin inhalation. This can decrease the cardiac output by 33% on average and further cause a reduction in the placental blood supply (55). Deficiency in placental blood supply may be associated with an increased risk of adverse maternal outcomes (12, 16, 56–58). Also, as we knew, snoring was a typical mild symptom of OSA. Patients with OSA were supposed to encounter a similar or even greater risk of adverse maternal–fetal outcomes than those with snoring alone. However, this trend was not distinctly observed in our result except for PTB. The influence of small size studies might be the cause or our assumption was not accurate.

Several limitations needed to be noted in this meta-analysis. To begin with, the results were possibly impacted by potential confounding factors. We extracted aORs from included study when they were available and calculated rORs when only raw data was accessible. The aORs provided by the previous studies were results after adjustments of confounding factors, while the rORs that we obtained were exposed to the influence of confounding factors. Since the risk factors may interact with SDB, thereby they may influence the risk of pregnancy complications or adverse maternal outcome in a different way. The most noteworthy confounding factor was obesity. As admitted by ICSD-3, snoring is linked to obesity and the major predisposing factor for OSA is excess body weight. However, OSA and obesity commonly coexist and even have similar clinical consequences such as insulin resistance and oxidative stress. Thus, it was a huge challenge to separate the effects of OSA from obesity. Moreover, trials in this field were not sufficient enough to get a convincing result. In addition, some other factors which may also have great impact such as chemo sensitivity and ethnicity are not fully considered. Sleep apnea prevalence is known to be different among various ethnicities. For instance, OSA mechanisms might vary across ethnicities according to a prior study (59). Yet only one trial of 35 included studies pointed out that the ethnic difference has been adjusted, which renders our research lack consideration of ethnic difference. In summary, our meta-analysis provided a comprehensive review for the association of SDB and adverse pregnancy complications or maternal–fetal outcomes. SDB during pregnancy was significantly associated with an increased risk of GDM, PIH, PEC, and PTB, but not with fetal maternal outcomes (APGAR and BW). Respectively, OSA was linked with an increased risk of GDM, PIH, PEC, and PTB, snoring alone appeared to increase the risk of GDM, PIH, and PEC. The result from our study provided new insights into maternal–fetal health care and leaded us to carry out ongoing research in this area.

## Author Contributions

LL, KZ, and JH: substantial contribution to the conception and design of the work; acquisition, analysis, and interpretation of the data; drafting the manuscript and revising it critically for important intellectual content; final approval of the version to be published; agreement to be accountable for the work. SL: interpretation of the data; critical revision of the manuscript; final approval of the version to be published; agreement to be accountable for the work.

## Conflict of Interest Statement

The authors declare that the research was conducted in the absence of any commercial or financial relationships that could be construed as a potential conflict of interest.

## Supplementary Material

Figure S1 Influence analysis of the included studies under GDM, PIH, and PEC. GDM, gestational diabetes mellitus; PIH, pregnancy-induced hypertension; PEC, preeclampsia.

Figure S2 Influence analysis of the included studies under BW, APGAR, and PTB. BW, birth weight; APGAR, APGAR score; PTB, preterm birth.

Figure S3 Publication bias analysis of the included studies under GDM, PIH, and PEC. GDM, gestational diabetes mellitus; PIH, pregnancy-induced hypertension; PEC, preeclampsia.

Figure S4 Publication analysis of the included studies under BW, APGAR, and PTB. BW, birth weight; APGAR, APGAR score; PTB, preterm birth.

Figure S5 Funnel plot of the included studies under BW, APGAR, PTB, GDM, PIH, and PEC. BW, birth weight; APGAR, APGAR score; PTB, preterm birth; GDM, gestational diabetes mellitus; PIH, pregnancy-induced hypertension; PEC, preeclampsia.

## Abbreviations

SDB, sleep-disordered breathing; SRBDs, sleep-related breathing disorders; OSA, obstructive sleep apnea; GDM, gestational diabetes mellitus; PIH, pregnancy-induced hypertension; PEC, preeclampsia; PTB, preterm birth; BW, birth weight; BMI, body mass index; aORs, adjusted odds ratios; CI, confidence interval; rORs, raw odds ratios; ORs, odds ratios; WMDs, weighted mean differences.
