# Supplementary figures and images for: Association between Sleep-Disordered Breathing during Pregnancy and Maternal and Fetal Outcomes: An Updated Systematic Review and Meta-Analysis
Source: Front Neurol. 2018 May 28;9:91. doi: 10.3389/fneur.2018.00091 (PMC5985400; doi:10.3389/fneur.2018.00091)

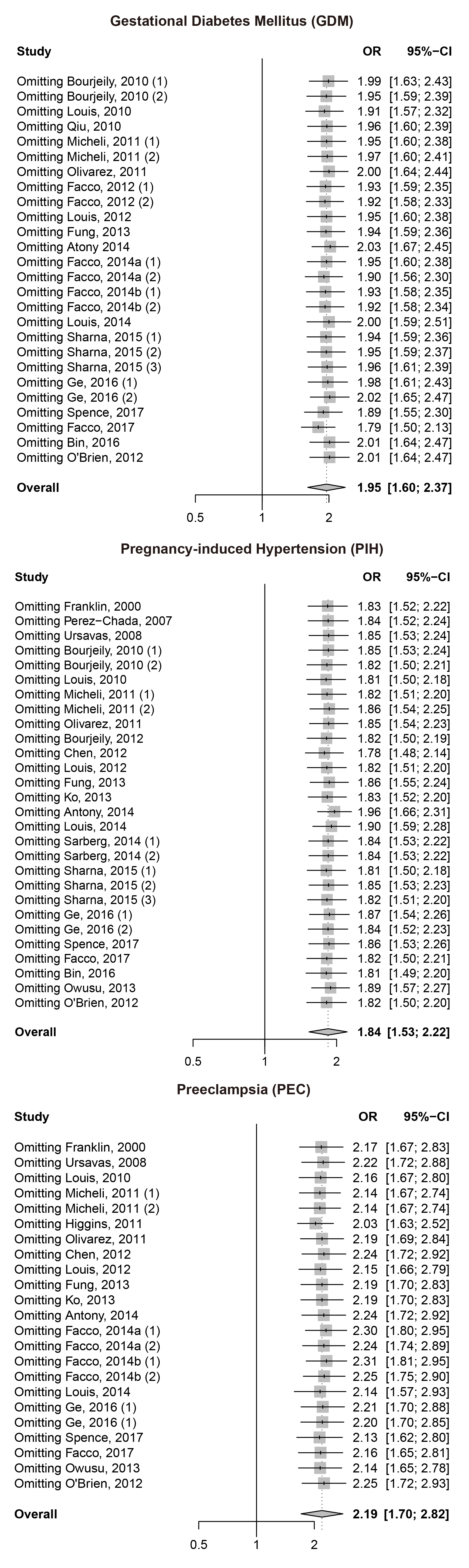

Supplement: Figure S1 — Influence analysis of the included studies under GDM, PIH, and PEC. GDM, gestational diabetes mellitus; PIH, pregnancy-induced hypertension; PEC, preeclampsia. [file image_1.jpeg]

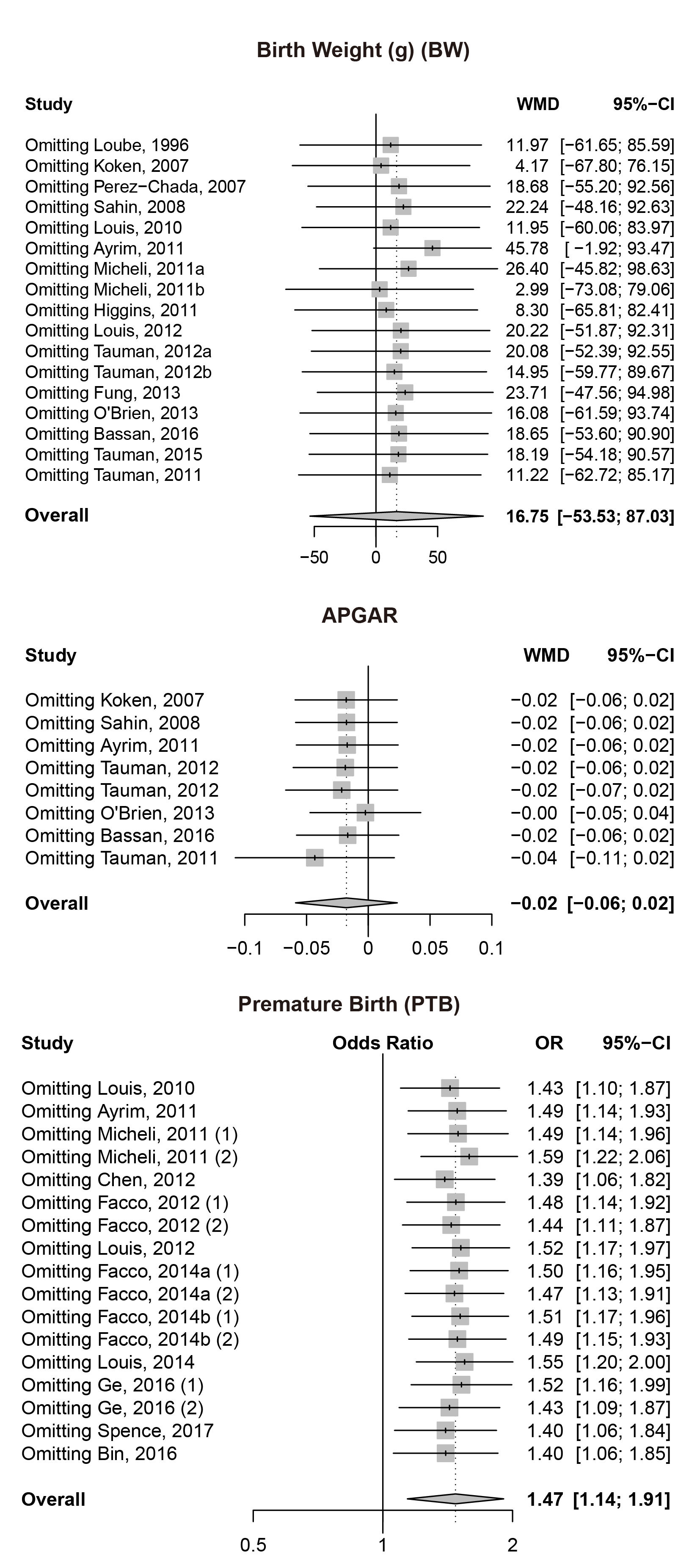

Supplement: Figure S2 — Influence analysis of the included studies under BW, APGAR, and PTB. BW, birth weight; APGAR, APGAR score; PTB, preterm birth. [file image_2.jpeg]

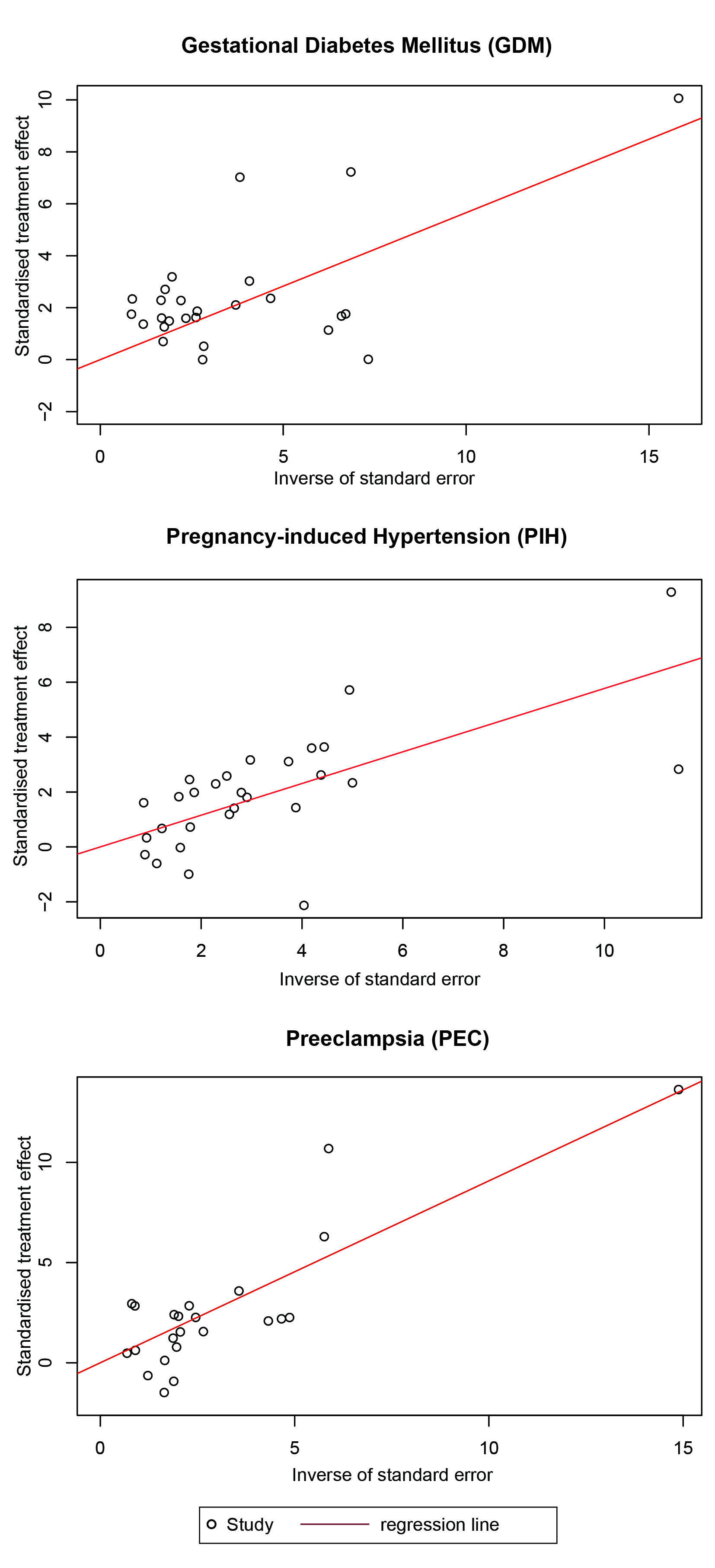

Supplement: Figure S3 — Publication bias analysis of the included studies under GDM, PIH, and PEC. GDM, gestational diabetes mellitus; PIH, pregnancy-induced hypertension; PEC, preeclampsia. [file image_3.jpeg]

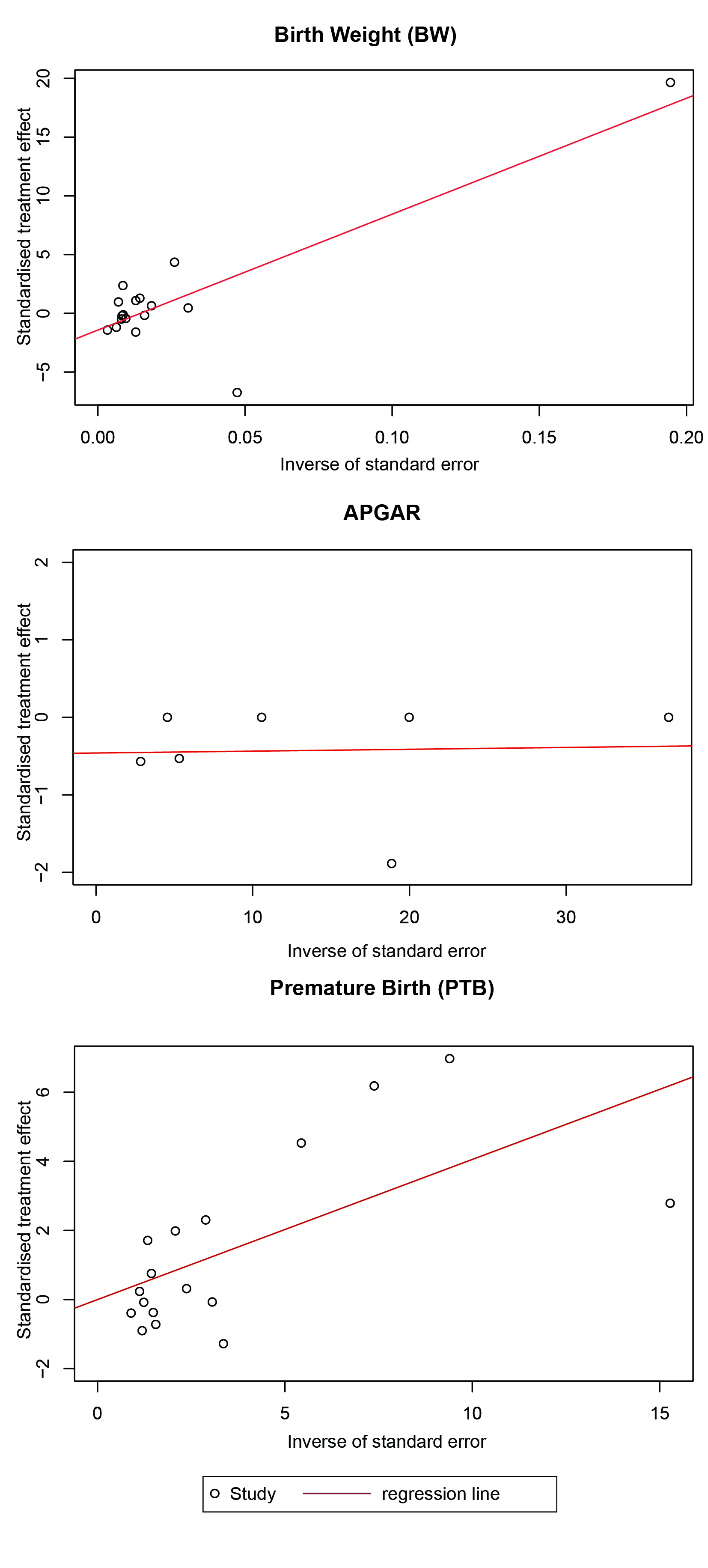

Supplement: Figure S4 — Publication analysis of the included studies under BW, APGAR, and PTB. BW, birth weight; APGAR, APGAR score; PTB, preterm birth. [file image_4.jpeg]

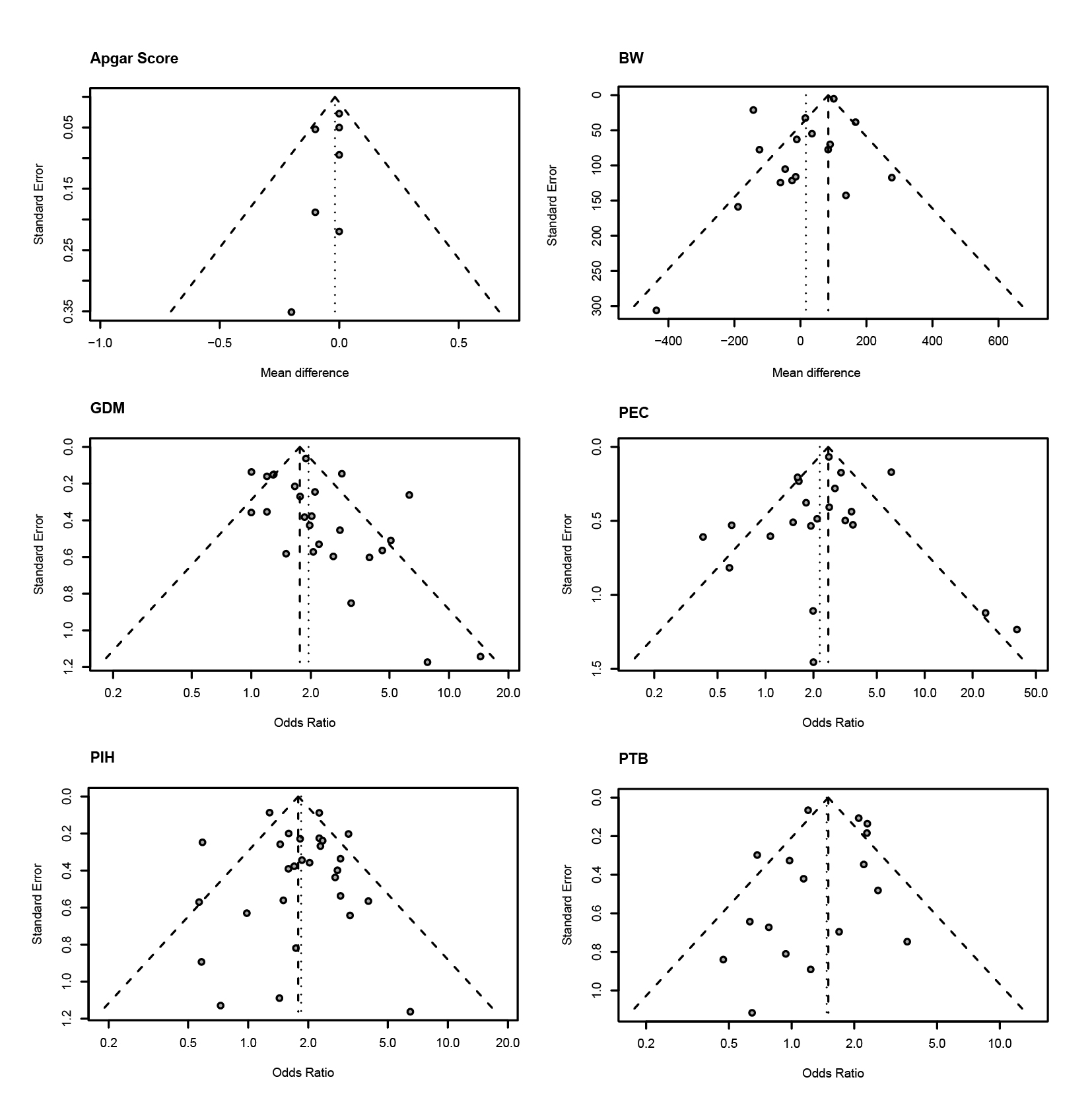

Supplement: Figure S5 — Funnel plot of the included studies under BW, APGAR, PTB, GDM, PIH, and PEC. BW, birth weight; APGAR, APGAR score; PTB, preterm birth; GDM, gestational diabetes mellitus; PIH, pregnancy-induced hypertension; PEC, preeclampsia. [file image_5.jpeg]
